# Impact of the source and serial passaging of goat mesenchymal stem cells on osteogenic differentiation potential: implications for bone tissue engineering

**DOI:** 10.1186/s40104-016-0074-z

**Published:** 2016-03-05

**Authors:** Hoda Elkhenany, Lisa Amelse, Marc Caldwell, Ramadan Abdelwahed, Madhu Dhar

**Affiliations:** Department of Large Animal Clinical Sciences, University of Tennessee, Knoxville, TN 37996 USA; Department of Surgery, Faculty of Veterinary Medicine, Alexandria University, Edfina, Behera, 22785 Egypt

**Keywords:** Caprine mesenchymal, Osteogenesis stem cells

## Abstract

**Background:**

Adult mesenchymal stem cells (MSCs) can be conveniently sampled from bone marrow, peripheral blood, muscle, adipose and connective tissue, harvested from various species, including, rodents, dogs, cats, horses, sheep, goats and human beings. The MSCs isolated from adult tissues vary in their morphological and functional properties. These variations are further complicated when cells are expanded by passaging in culture. These differences and changes in MSCs must be considered prior to their application in the clinic or in a basic research study. Goats are commonly used as animal models for bone tissue engineering to test the potential of stem cells for bone regeneration. As a result, goat MSCs isolated from bone marrow or adipose tissue should be evaluated using *in vitro* assays, prior to their application in a tissue engineering project.

**Results:**

In this study, we compared the stem cell properties of MSCs isolated from goat bone marrow and adipose tissue. We used quantitative and qualitative assays with a focus on osteogenesis, including, colony forming unit, rate of cell proliferation, tri-lineage differentiation and expression profiling of key signal transduction proteins to compare MSCs from low and high passages. Primary cultures generated from each source displayed the stem cell characteristics, with variations in their osteogenic potentials. Most importantly, low passaged bone marrow MSCs displayed a significantly higher and superior osteogenic potential, and hence, will be the preferred choice for bone tissue engineering in future *in vivo* experiments. In the bone marrow MSCs, this process is potentially mediated by the p38 MAPK pathway. On the other hand, osteogenic differentiation in the adipose tissue MSCs may involve the p44/42 MAPK pathway.

**Conclusions:**

Based on these data, we can conclude that bone marrow and fat-derived MSCs undergo osteogenesis via two distinct signaling pathways. Even though the bone marrow MSCs are the preferred source for bone tissue engineering, the adipose tissue MSCs are an attractive alternative source and undergo osteo-differentiation differently from the bone marrow MSCs and hence, might require a cell-based enhancer/inducer to improve their osteogenic regenerative capacity.

## Background

There has been an explosion of research publications in the field of mesenchymal stem cells (MSCs) in the past 10 yr. Many researchers have sought to exploit their potential as a source of reparative cells for clinical use in a variety of contexts [1]. Adult stem cells are multipotent, undifferentiated, self-renewing capable of healing and regeneration of injured tissues [2–4]. Adult MSCs were initially identified in bone marrow (BM) and since then have been isolated and characterized from different human and animal adult tissues, peripheral blood, adipose, muscle, skin, dental pulp and other tissues [5–10].

Adult stem cells can be conveniently sampled from an extensive array of sources bypassing the ethical controversy associated with the embryonic stem cells, which is increasingly affecting the use of stem cell research and therapy. Even though, MSCs can be isolated from a variety of tissues and species, there is significant variation in their biological properties (morphological and functional changes) [11–17]. These changes can be due to the source of MSCs, age-dependent donor-to-donor variation, and due to the signaling pathways that each cell type undergoes during differentiation. These variations are further complicated when cells are expanded by passaging in culture. Cell cultures generated from MSCs are not immortalized cell lines but are primary cultures which have limited lifespan and they undergo the process of senescence and stop dividing after a certain number of population doublings while generally retaining their viability [18]. These are inherent challenges associated with the *in vitro* expansion of primary cultures of MSCs. As cells divide in culture, they grow and fill the available area or volume and reach confluency when the given area is filled. If the cells become over-confluent, cell-to-cell contact can stimulate cell cycle arrest, causing cells to stop dividing, cell-to-cell contact can stimulate cellular differentiation, and genetic and epigenetic alterations can occur, potentially leading to overgrowth of abnormal, culture-adapted cells with decreased differentiation and increased proliferative capacity [19]. All these cell culture issues can directly or indirectly affect the biological function of cells obtained during passaging. Passaging of MSCs is important and necessary to obtain enough cell numbers for clinical applications. Hence, for controlled animal model experiments, we need to investigate and compare the properties of MSCs from different sources at harvest and during passaging, prior to their use in these experiments.

The most extensively used sources of undifferentiated MSCs are bone marrow and adipose tissue. Bone marrow aspirates contain only small proportion of MSCs and their number and differentiation capacity decrease with age of donors [20]. While, adipose tissue is abundant, relatively easy to access from the body and the cell numbers do not decrease with the age [6].

Goats are widely used as large animal preclinical model for bone tissue engineering. Goats have a body weight similar to that of human beings, which mimics the same load bearing factors on human fracture. The metabolic rate of goats is similar to that of humans and most importantly, goats have a body size suitable for implantation of implants and prostheses [21–24]. The focus of our research is bone tissue engineering, hence, in this study, experiments were designed to compare and assess the cell culture properties of bone marrow- and fat-derived goat MSCs and to investigate whether these MSCs undergo any biological changes when they are expanded in culture. This comparison would then be used to identify the optimal source of goat MSCs for bone tissue engineering. We hypothesize that MSCs derived from bone marrow and adipose tissue differ in their proliferation and osteogenic potentials which may be affected at multiple levels. To prove our hypothesis, we compared goat/caprine bone marrow - derived MSCs from low (cBMMSCs^low^), and high (cBMMSCs^high^) passages to the adipose - derived MSCs from identical passage numbers (cADMSCs^low^ and cADMSCs^high^). Comparison was made by assessing their proliferation capacity, trilineage differentiation and expression profile of osteogenic specific proteins.

## Methods

### Media and chemicals

All chemicals, cell culture supplements, and disposable tissue culture supplies were purchased from Thermo Fisher Scientific (Fisher Scientific, Pittsburgh, PA) unless otherwise noted. Animal experimental procedures were carried out according to protocols approved by the Institutional Animal Care and Use Committee.

### Tissue collection and isolation of MSCs

Goat adipose-derived MSCs were isolated and expanded in tissue culture using methods previously reported for equine and goat fat [11, 25]. Briefly, 5 g of subQ adipose tissue was harvested from the inguinal area of a 2 - year - old mixed breed goat. Subsequently, the tissue was rinsed with a 10 % bleaching solution for disinfection and, after washing thoroughly with 1× PBS, was minced into small pieces. Tissue was then digested in a mixture of 2 mg/mL collagenase (Type I; 260U, and type IV; 220U) solution in 1× PBS with continuous shaking at 37 °C for 40 min. The digested sample was filtered through a 100 micron cell strainer and centrifuged at 800 *g* for 10 min at 10 °C, resulting in a stromal vascular fraction (SVF) pellet. The pellet was resuspended in medium consisting of Dulbecco’s modified medium-F12, with 10 % fetal bovine serum and 100 I.U./mL penicillin, 100 μg/mL streptomycin (referred to as the growth medium). Roughly, 2–4 × 10^6^ cells/5 g of tissue were obtained and seeded in T75 culture flasks in growth media and allowed to adhere for 48 h. Stromal medium was changed every 3 d until the cells reached 80 % confluency. Adherent cADMSCs were passaged by digestion with 0.25 % trypsin/EDTA.

Previously isolated, characterized and cryopreserved goat bone marrow-derived MSCs were used in all experiments [26].

All experiments were carried out simultaneously using the cADMSCs and cBMMSCs. In all experiments, passages 2–3 were considered as the lower passage, and passage 12-14 was considered as higher passages. Hence, 4 cell types were investigated in this study. These are referred to as: cBMMSCs^low^, cBMMSCs^high^, cADMSCs^low^, and cADMSCs^high^.

### Colony forming unit (CFU) assays

Freshly isolated mononuclear cells present in the bone marrow buffy coat (Passage 0) and 1× 10^3^ cBMMSCs of passage 3 and passage 12 each were seeded in 100 mm tissue culture dishes. Similarly, a portion of digested fat remaining after collagenase digestion and representing passage 0 and 1× 10^3^ cADMSCs of passage 3 and passage 12 each were seeded in 100 mm tissue culture dishes. After incubation for 7 d in a 37 °C, 5 % CO_2_ incubator, cells were washed with PBS and fixed with 10 % formalin for 10 min at room temperature. Subsequently, the colonies were stained with 0.5 % crystal violet solution for 5 min at room temperature, and imaged using Zeiss Axiovert 40C microscope (Carl Zeiss MicroImaging, Inc., Thornwood, NY, USA) equipped with Nikon Digital Sight DS-Qi1Mc camera (Nikon Instruments Inc., Melville, NY, USA).

### Cell proliferation assay

Cells from cBMMSCs^low^, cBMMSCs^high^, cADMSCs^low^, and cADMSCs^high^ at density of 5×10^3^/well were seeded in 24 well tissue culture plates. Cell proliferation were measured at d2, d7 and d10 using CellTitre 96® Aqueous nonradioactive (MTS) assay (Promega, Madison, WI) according to the manufacturer’s instructions. Briefly, MTS reagent prepared in a 5:1 ratio relative to the media was added to the cells and incubated for 3 h at 37 °C and 5 % CO_2_. Then 100 μL (*n* = 3) was transferred to 96-well plate and the absorbance was measured at 490 nm. Non seeded wells with media only were used as blank.

### Tri-lineage differentiation assays

Osteogenesis, chondrogenesis, and adipogenesis were performed as described earlier with slight modifications [26]. Roughly 2×10^5^ cells were seeded in complete growth media, and 48 h later, when cells were 70 % confluent, lineage-specific differentiation was induced. Cells were exposed to specific differentiation media throughout the differentiation process and media was replenished every 3 d. For each differentiation assay, an identical number of non-induced cells (cells without any differentiation media) were used as controls. Tri-lineage differentiation was monitored and visualized using phase contrast microscopy and cell-specific staining. Alizarin red staining at d21, alcian blue staining at d14, and oil-red-o staining at d7, for osteogenesis, chondrogenesis, and adipogenesis, respectively were used. All images were obtained using a Zeiss Axiovert 40C microscope (Carl Zeiss MicroImaging, Inc., Thornwood, NY, USA) equipped with Nikon Digital Sight DS-Qi1Mc camera (Nikon Instruments Inc., Melville, NY, USA).

### Osteogenic and chondrogenic differentiation assays

Quantitative assays were carried out at d7, d14 and d21 using methods described earlier [27, 28]. For osteogenic quantitation, alizarin red was extracted using 10 % cetylpyridium chloride for 30 mins. and the color was detected at 550 nm. For chondrogenic quantitation, alcian blue was extracted using 6 mol/L guanidine hydrochloride for 24 h, and the color was detected at 620 nm.

### Western blot analysis

To obtain whole cell protein extracts, low and high passaged undifferentiated and osteo-differentiated cADMSCs and cBMMSCs at d7, d14 and d21 were collected in cell lysis buffer (Boston Bioproducts, Ashland, MA). Whole cell extracts were sonicated and centrifuged to obtain the total proteins. Total protein in each sample was quantitated and concentrations were obtained using the BCA assay (Pierce, Thermo Scientific, Rockford, IL).

For western blot analysis, 25 μg of proteins were electrophoretically separated by 10 % SDS-polyacrylamide gel electrophoresis and transferred to nitrocellulose membranes. The membranes were blocked with 5 % bovine serum albumin and incubated with 1–5 μg of primary antibodies against β-Tubulin (Santa Cruz, Dallas, Texas), osteopontin, (Abcam, Cambridge, MA), BMP-7, (Millipore, Darmstadt, Germany), p38 MAPK (Cell signaling, Danvers, MA), and p44/42 MAPK(Cell Signaling) proteins. Bound proteins were visualized with HRP-conjugated anti-rabbit IgG for β-Tubulin, osteopontin, p38 and p44/42, or anti-mouse IgG for BMP-7. Enhanced Chemiluminescence assay was used for the visualization of the protein bands. The intensity of each band was analyzed using Image J software (Image j 1.48 V, Wayne Rasband, National institutes of health, USA, http://imagej.nih.gov/ij, Java 1.6.0–20 (64-bit).

### Immunofluorescence (IF)

Undifferentiated and differentiated cells were stained with osteogenic-specific protein, osteopontin on d21. For staining, cells were fixed with 4 % paraformaldehyde, blocked with 5 % normal serum for 30 min at room temperature and finally incubated with 5 μg of anti-osteopontin (Abcam). Osteopontin was detected using Alexa Fluor 647 donkey anti-rabbit IgG. The cells were mounted with SlowFade Gold Antifade with DAPI reagent (thermo Fisher Scientific, Waltham, MA) and images were taken with a laser scanning spectral confocal microscope (Leica TCS SP2; Leica Microsystems©, Wetzlar, Germany).

### Statistics

All quantitative data are presented as mean ± standard deviation (SD) with no less than three replicates for each experimental condition. Comparison between experimental and control groups were performed with Factorial ANOVA with the use of SAS Enterprise Guide 6_1. A value of *P* <0.05 was considered statistically significant.

## Results and discussion

### Stem cell characteristics

Adult mesenchymal stem cells were isolated from goat (caprine) subQ fat and sternal bone marrow aspirate. Cells isolated from each of these sources were characterized by assessing their *in vitro* adherence, proliferation, and tri-lineage differentiation profiles. Each of these characteristics were compared between cBMMSCs^low^, cBMMSCs^high^, cADMSCs^low^, and cADMSCs^high^.

1. Colony forming unit (CFU) and Cell proliferation assay

Our results confirmed that the cells from the mononuclear fraction of goat bone marrow and the SVF from goat subQ fat contain the mesenchymal progenitor cells which are capable of adherence to tissue culture surface, proliferation to form a cluster of colonies and providing sufficient number of cells within 7 d. These properties were maintained even at higher passages. However, some striking differences were observed.

**Fig. 1:**
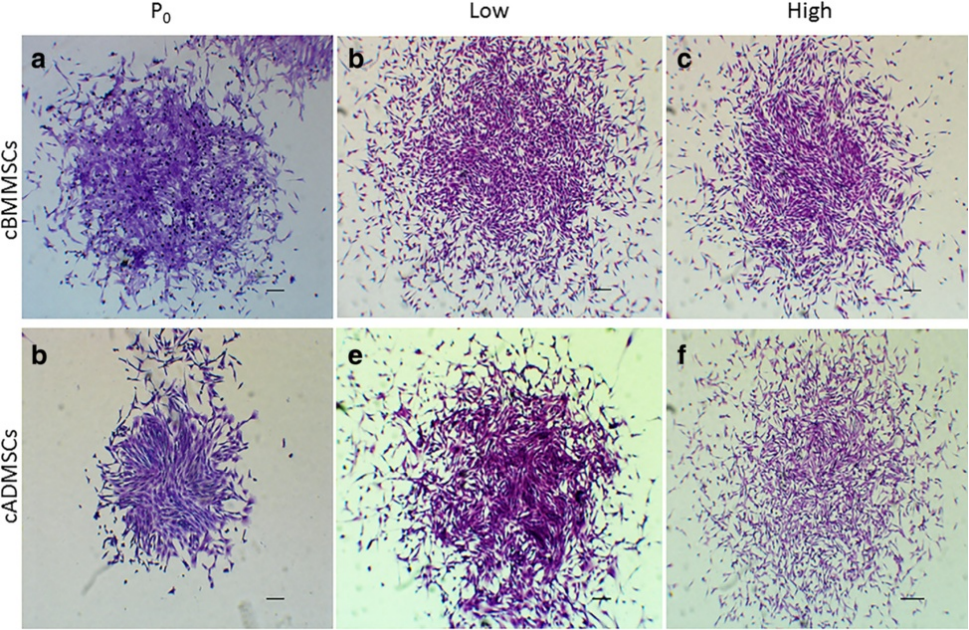
Colony forming assay. A representative photograph of **a** freshly isolated bone marrow-derived mononuclear cells (passage 0) **b** cBMMSCs passage 3 **c** cBMMSCs passage 13 **d** Stromal vascular fraction (SVF) of adipose tissue **e** cADMSCs passage 3 and **f** cADMSCs passage 11. Clusters of colonies were visualized by crystal violet staining. Scale bar = 100 μm

**Fig. 2:**
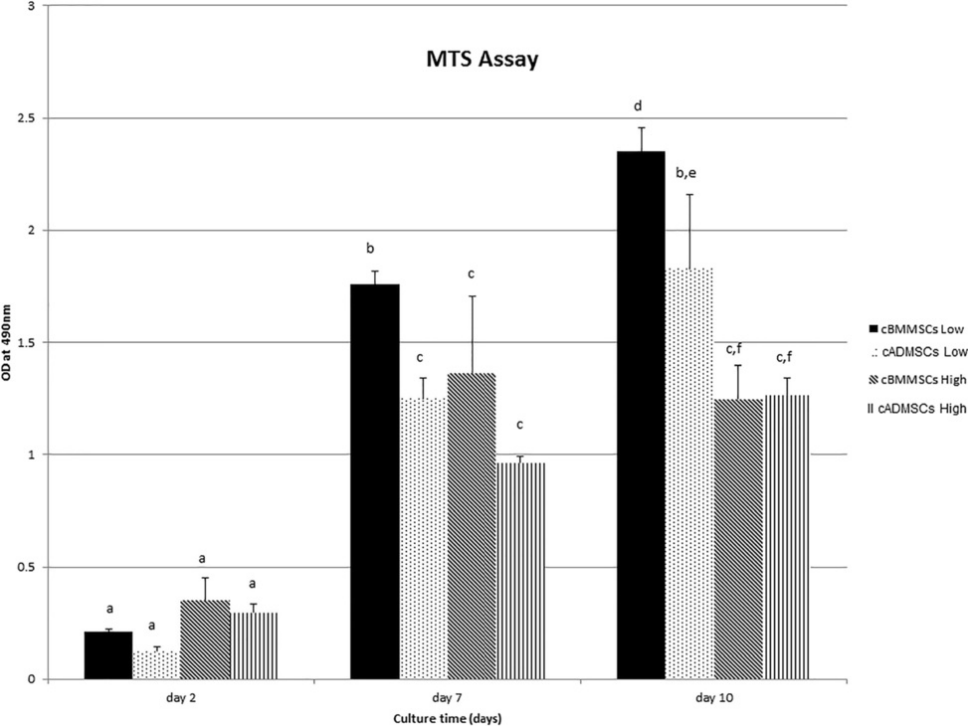
MTS assay. Comparison of proliferation of cBMMSCs and cADMSCs low and high passage over the period of 10 days. Significantly different values (*P* < 0.05) are indicated by superscript letters. Identical letters indicate no significance

Subjective evaluation using CFU assay revealed that all 4 MSC cultures were capable of growing in clusters (Fig. 1). Cell viability and proliferation as judged by the MTS assay however, showed significant differences (Fig. 2). Both the cBMMSCs^low^ and cADMSCs^low^ proliferated over the course of 10 d and cell numbers increased 11–15 fold significantly with time. Specifically, the cBMMSCs^low^ and cADMSCs^low^ demonstrated a significantly higher rate of proliferation between d2 and d10. Additionally, as judged by the regression analyses, there was a linear increase in proliferation in cBMMSCs^low^ and cADMSCs^low^ only in this time period. This pattern however, was not maintained in the higher passaged cells. Cells from cBMMSCs^high^ and cADMSCs^high^ did not proliferate linearly over the same time period, cell numbers increased roughly 3–5 folds only, and there was no significant difference between their rates of proliferation. The difference in proliferation between cBMMSCs and cADMSCs is similar to that reported earlier in human [17] and sheep [29] studies, however, it disagrees with the data reported by Zhu et al who demonstrated that human ADMSCs proliferate higher than BMMSCs [30]. The comparison between passage numbers is unique to our study.

2- Tri-lineage differentiation assays

**Fig. 3:**
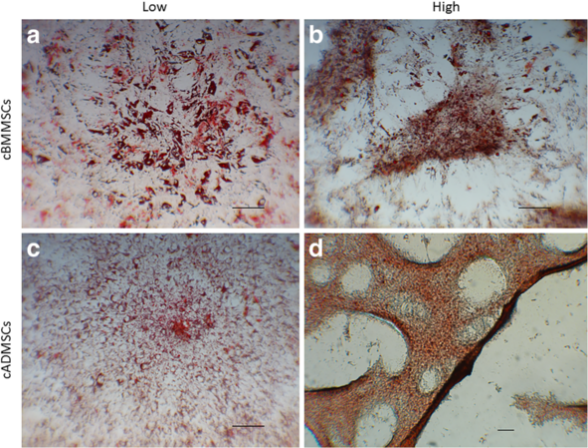
Adipogenesis differentiation. Comparison of cBMMSCs and cADMSCs, undergoing adipogensis as detected by the formation of neutral lipid vacuoles stained with Oil Red-O at 21 days after induction **a** cBMMSCs^Low^ 10X **b** cBMMSCs^High^ 10X **c** cADMSCs^Low^ 10X **d** cADMSCs^High^ 5X

**Fig. 4:**
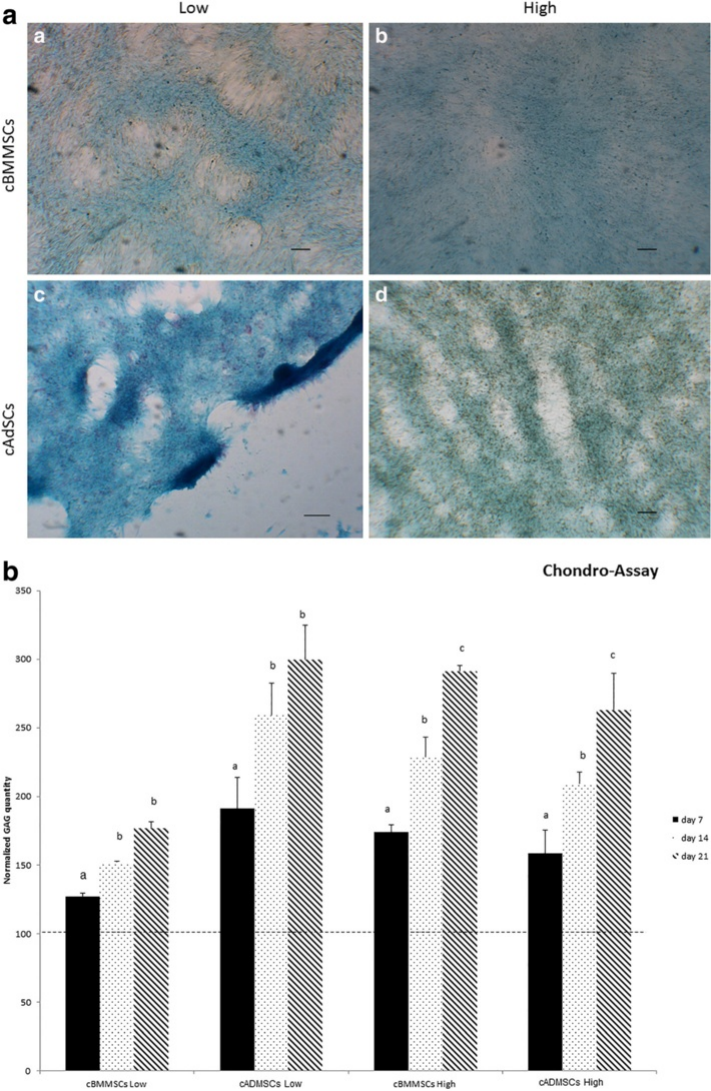
Chondrogenesis differentiation. Comparison of cBMMSCs and cADMSCs. **a** chondrogenesis as detected by alcian blue staining at d 14 after induction (*a*) cBMMSCs^Low^, 5X (*b*) cBMMSCs^High^, 5X (*c*) cADMSCs^Low^, 5X (*d*) cADMSCs^High^, 5X. **b** Graph representing normalized alcian blue staining (indicating the GAG content in the chondrocyte differentiated cBMMSCs and cADMSCs from low and high passages). Dotted line indicates the GAG content in undifferentiated cells normalized to 100. Significantly different values (*P* < 0.05) represent the comparison between the three time points within a specific group. Identical letters indicate no significance

**Fig. 5:**
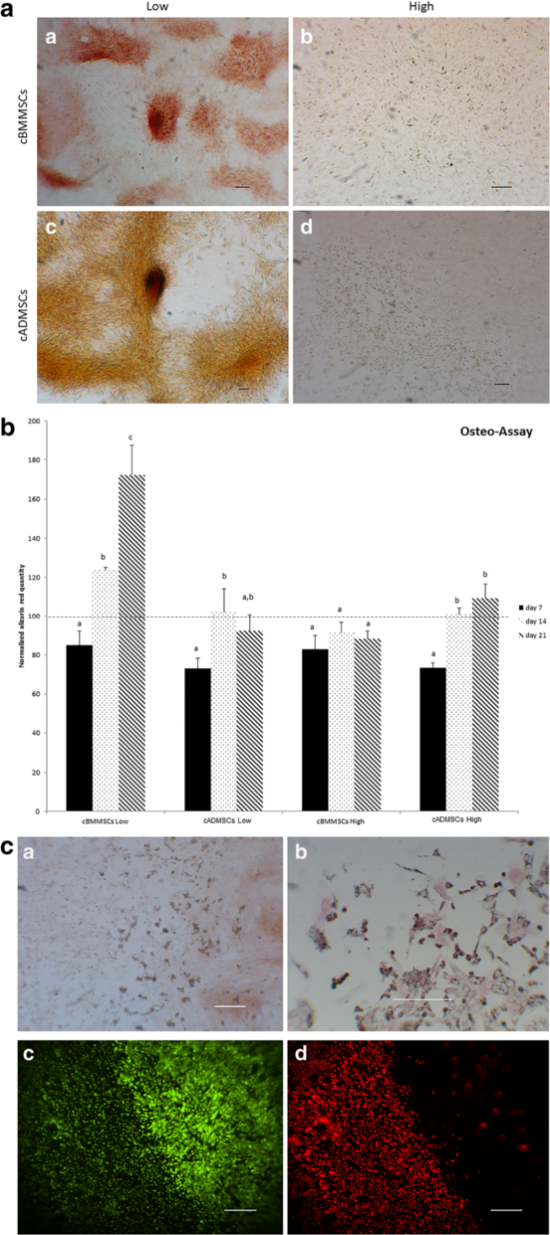
**a** Osteogenesis differentiation. Comparison of cBMMSCs and cADMSCs. Osteogenesis as detected by the formation of mineralized nodules stained with alizarin red at d 21 after induction (*a*) cBMMSCs^Low^, 5X (*b*) cBMMSCs^High^, 5X (*c*) cADMSCs^Low^, 5X (*d*) cADMSCs^High^, 5X. **b** Graph representing normalized alizarin red staining (indicating the calcium content in the mineralized nodules in the osteocyte differentiated cBMMSCs and cADMSCs from low and high passages). Dotted line indicates the alizarin content in undifferentiated cells normalized to 100. Significantly different values (*P* < 0.05) represent the comparison between the three time points within a specific group. Identical letters indicate no significance. **c** The cADMSCs^high^ have a tendency to undergo adipogenesis and cell senescence in presence of osteogenic differentiation medium. (*a*) Alizarin red staining – note the lack of staining and mineralized nodules, 10X (*b*) Oil Red-O staining – note the staining in a focused area during osteogenesis, 20X (*c*) Calcein am, – note the bright green fluorescence demonstrating viable cells, 10X (d) Propidium iodide – note the red fluorescence demonstrating the dead cells, 10X

Low and high passaged MSCs from both sources underwent tri-lineage differentiation, with some morphological differences (Figs. 3, 4 and 5).

#### Adipogenic differentiation

The cBMMSCs^low^ demonstrated clusters of large fat oil-red-o positive droplets, whereas, cADMSCs^low^ exhibited relatively small and dispersed droplets. The pattern of adipogenic differentiation in the high passaged cells from both sources was intriguing. Even though the cells were induced towards adipogenic differentiation, the differentiated cells appeared morphologically similar to chondrocytes (Fig. 3b & d). Typically, when MSCs are submitted to chondrogenic conditions in monolayer culture, they begin to condensate in response to the stimulus and form high-density three-dimensional cell aggregates [31]. Subjectively, a cell aggregate--like pattern described above and suggesting chondrogenesis was observed in the cADMSC^high^ cells. This observation is supported by previous studies [32, 33]. Nujib Ullah et al. showed that adipogenic ECM was positive for chondrocyte-specific proteins, collagen I, type II and IV, and also, demonstrated alkaline phosphatase activity indicating the ossified nature [33].

#### Chondrogenic differentiation

Subjective evaluation of chondrogenic differentiation (Fig. 4a) in all cell lines, exhibited similar patterns of differentiation. However significant differences, were observed in the level of chondrogenesis as measured by the GAG contents (Fig. 4b). The accumulation of GAGs significantly increased with time for each of the 4 cell lines, suggesting that cells from all sources had the potential to undergo chondrogenesis. There were no significant differences within the different cell sources; differences however, were significant between the passage numbers. For instance, cBMMSCs^low^ and cADMSCs^low^ both showed a significant increase in GAG content at d 14 (*P* < 0.05). The cBMMSCs^high^ and cADMSCs^high,^ however, showed a significant increase at d 21 (*P* < 0.05), suggesting delayed chondrogenesis in high passaged cells. These findings are supported by previous studies which report that the chondrogenic potential of canine and mouse MSCs is dependent on the age of the donor and the serial passaging of cells in culture [20, 34].

Results presented above demonstrate that the primary cultures of goat BM and ADMSCs undergo changes when they are passaged in culture, and these changes affect their proliferation, adipogenesis and chondrogenesis differentiations. Further investigations are required to fully understand these changes, but, are beyond the focus of this paper.

#### Osteogenic differentiation

Since, the focus of this study is to use goat as a preclinical model of bone tissue engineering, detailed experiments to assess the process and mechanism of osteogenesis differentiation in the four MSC cultures were carried out (Fig. 5). Subjective evaluation of osteogenic differentiation (Fig. 5a), exhibited significant differences in alizarin red staining in all cell lines. Presence of alizarin red stained mineralized nodules coupled with a significant increase in the calcium content (Fig. 5b), demonstrates that low passaged bone marrow-derived MSCs have the highest potential to undergo *in vitro* osteogenesis. This data is supported by the *in vivo* findings reported by Niemeyer et al and Xie et al., that the bone healing potential of BMMSCs was higher than the ADMSCs in sheep [35] and in rabbit [36].

The effect of serial passaging of cells on the osteogenic differentiation is unique to our study. When the cBMMSCs^high^ and cADMSCs^high^ were induced to undergo osteogenic differentiation, the cells did differentiate into osteocytes but, could not form mineralized nodules, suggesting a poor potential for differentiation. This was confirmed by the significantly low alizarin red content and the lack of mineralized nodules in the alizarin red staining (Fig. 5*a* and; *b*).

Another intriguing observation was that the cADMSCs^high^ exhibited adipocyte morphology when they were induced to differentiate into osteocytes, indicating a change in their property on passaging. Additionally, as judged by the presence of red fluorescence when stained with propidium iodide, majority of the differentiated cells were not viable (Fig. 5*c*). The results suggest that on passaging the cADMSCs^high^ might lose their potential for osteogenesis and cannot survive the presence of exogenously added growth factors. Interestingly, the oil-red-o staining in these cells demonstrated that the cells had undergone adipogenesis (Fig. 5c (*b*)). These results are in agreement with the data reported by Jaiswal et al. that when osteogenic differentiation is blocked using MAPK signaling, stem cells undergo adipogenic differentiation [37]. The exact mechanism of all these changes in the higher passaged cells is not known. We can only deduce that on culturing the cADMSCs undergo major morphological changes which can potentially affect their function and hence, should be considered prior to their use. Finer analysis revealed that the osteogenic potential of fat-derived MSCs is considerably reduced after 3–4 passages, whereas, bone marrow-derived MSCs retained this potential up to passage 6.

Taken together, even though MSCs from all 4 sources proliferate and undergo tri-lineage differentiation, the low passaged bone marrow-derived MSCs described in this study exhibit properties that present them as the optimal source of MSCs for bone tissue engineering.

#### Mechanism of osteogenesis

The precise cellular signaling mechanisms by which bone synthesis is controlled within the progenitor cells remains undefined. The process more likely includes early electrophysiological responses, possibly mediated by intra– and extra– cellular signaling pathways, i.e. regulated by transcription factors at the transcriptional level and/or expression of key proteins at the translational level. All these processes can thus regulate the osteoblast phenotype [38]. In order to assess the mechanism of osteogenesis in each of the four MSC primary cultures generated in this study, a combination of immunofluorescence and immunoblotting analyses were used and the expressions of key target proteins, including, osteopontin, p38, p44/42 and BMP-7, known to have major biological roles in specific phases of the osteogenesis process were investigated. Our goal was to try and understand what makes one MSC culture better suited for osteogenesis compared to the other. Understanding the mechanism and identifying the key molecular and cellular factors of osteogenesis expressed in bone marrow or fat MSCs, may be useful for developing new treatment strategies to improve the osteogenic potential of MSCs which in turn will accelerate bone formation, necessary for bone tissue engineering.

Osteopontin (OPN), is a protein expressed in the bone-related matrix and is generally considered to be a specific marker expressed in the late stage of osteogenic differentiation [39–41]. Hence, OPN is expressed in cells with a potential to undergo osteogenesis, and its expression should increase with differentiation [42].

**Fig. 6:**
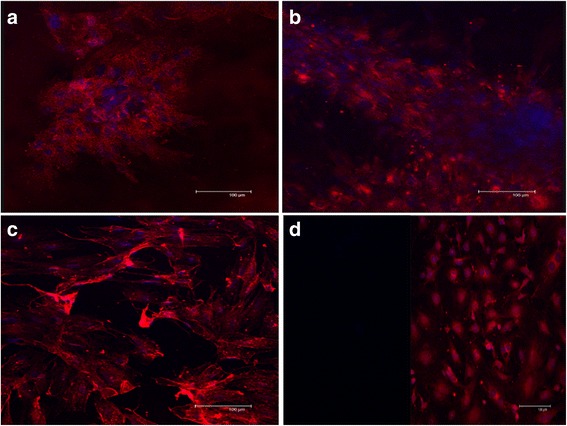
Immunofluorescence analyses. Detection of osteogenic specific markers in cBMMSCs and cADMSCs. Immunofluorescence showing the expression of OPN in **a** cBMMSCs^Low^, 20X **b** cADMSCs^Low^, 20X **c** cBMMSCs^High^, 20X **d** cADMSCs^High^, 20X

**Fig. 7:**
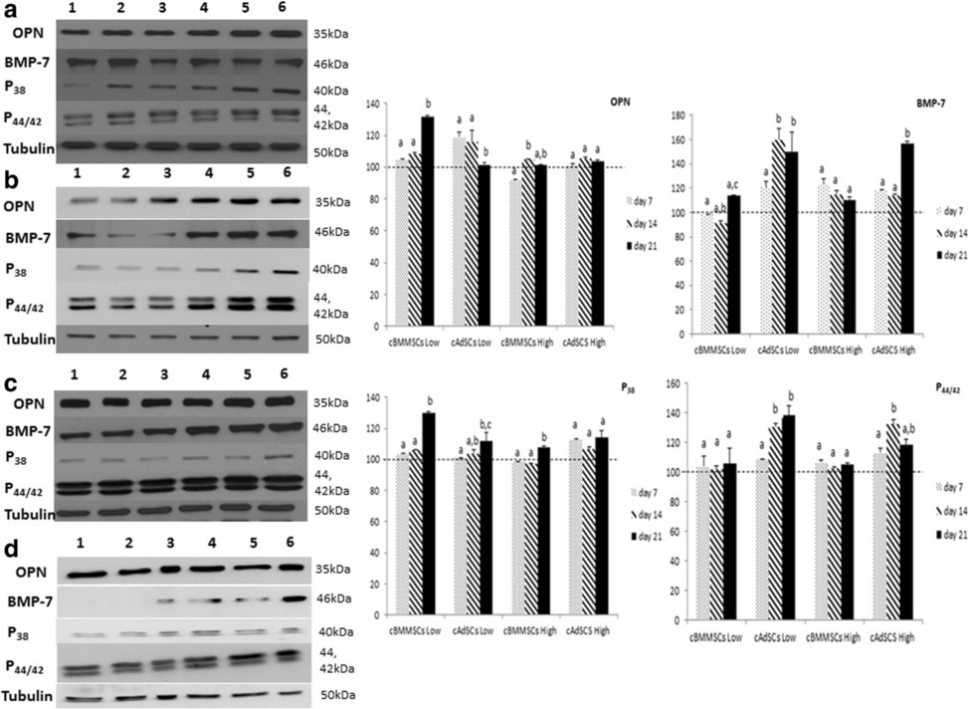
Immunoblot analyses. Western blots showing the expression of OPN, BMP-7, p38 and p44/42 in low and high passaged cBMMSCs and cADMSCs. Autoradiographs of undifferentiated (lanes 1, 2, 3) and differentiated (lanes 4, 5, 6) cells at three time points, d 7, 14, 21 are shown. Graphs represent the relative expression of each protein in the differentiated samples normalized with that in the undifferentiated samples. β tubulin was used as a housekeeping control. Dotted line indicates the protein expression in undifferentiated cells normalized to 100. Significantly different values (*P* < 0.05) represent the comparison between the three time points within a specific group. Identical letters indicate no significance

Results in Figures 6 and 7 show the expression of OPN in undifferentiated and differentiated MSCs from all sources, suggesting that MSCs have the potential to undergo osteogenesis. Significant up-regulation of OPN and significant increase in the alizarin red content (Figs. 5b & 6) in differentiated cBMMSCs^low^ data confirms that the cBMMSCs ^low^ have the highest potential to undergo osteogenesis. The expression level of OPN protein can be considered as a good indicator of osteogenic differentiation potential. This pattern of expression is further confirmed by the quantitative osteogenic assay.

Mitogen-activated protein kinase (MAPK) pathway is a chain of proteins in the cell that communicates a signal from a receptor on the surface of the cell to the DNA in the nucleus. The MAPK signaling pathway comprises of a cascade of three protein kinases: extracellular signal-regulated kinase (ERK), p38, and c-Jun NH2-terminal kinase (JNK), and control a variety of important cellular events, including, cell proliferation, apoptosis and differentiation [43–46]. The exact role of MAPK proteins in the osteogenesis process is unclear. Several reports suggest their pro – or the anti- effect on this process [43–50]. Of relevance to this study, recent findings suggest that the MAPK pathway has a regulatory role in the osteogenesis process. The JNK and ERK pathways are repressors of osteogenesis, whereas p38 pathway is an enhancer of osteogenesis of BMMSCs [47, 48, 50].

Western blot analysis data showed that both the p38 and OPN proteins were significantly up-regulated on d21 when cBMMSCs^low^ differentiated into osteocytes, suggesting that the osteogenic differentiation in these cells is mediated by p38 and not p44/42 proteins (Fig. 7). These findings are in agreement with some of the previous studies [48, 50], which reported that blocking of ERK pathway promoted ALP activity and mineralization. This pattern was however, not observed in the cBMMSCs^high^_._

Comparatively, p44/42 was significantly up-regulated in cADMSCs^low and high^ suggesting its involvement in osteogenesis of adipose – derived MSCs (Fig. 7). Additionally, bone morphogenetic protein 7 (BMP-7), another protein known to have a role in osteo-differentiation [51, 52] was significantly up-regulated in cADMSCs ^low and high^, while its expression in cBMMSCs^low and high^ were significantly lower than in the cADMSCs. Results suggest that BMP-7 may have a role in osteogenic differentiation in fat-derived MSCs and not bone marrow – derived MSCs. The exact role of BMP-7 is controversial. Knippenberg et al. demonstrated that BMP-7 is involved in the chondrogenesis of adipose-derived MSCs; whereas, Katja et al. reported that BMP7 is required in the adipogenic differentiation of bone marrow MSCs [53, 54].

In summary, all data suggest that the MSC population of cells cultured from goat bone marrow and fat have the potential to undergo osteogenic differentiation *in vitro* under optimal culture conditions. The MSCs from both sources undergo changes during passaging, the exact mechanism of which is still unclear. Results presented in this study demonstrate that the process of osteogenic differentiation of MSCs, is mediated by different protein factors in bone marrow and adipose tissue. As judged by the OPN expression and quantitatively confirmed by the osteogenic assay, the cBMMSCs^low^ osteogenesis is probably mediated by p38. On the other hand, osteogenic differentiation in cADMSCs^low^ is mediated via p44/42 and BMP-7 signaling. Differences in signaling during differentiation can impact their function.

**Fig. 8:**
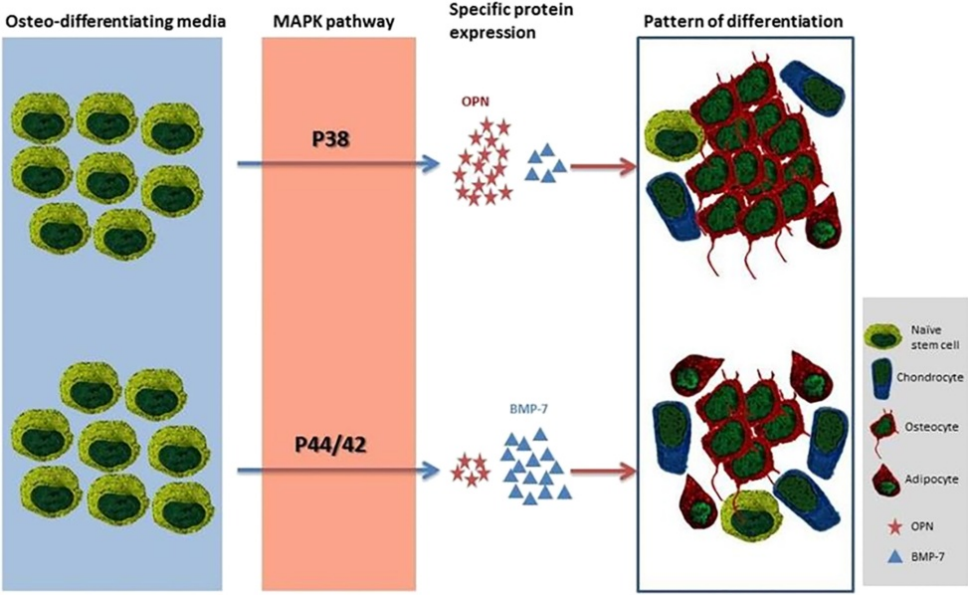
Schematic representation of the proposed mechanism of osteogenesis. Key targets involved and potential signaling networks regulating the osteogenesis of cBMMSCs and cADMSCs

Based on our results, we propose that MSCs from either the bone marrow or the adipose tissue are naïve, undifferentiated stem cells which have the potential to differentiate into osteocytes, chondrocytes and adipocytes. When they are exposed to the osteogenic signals, the cells have the potential to undergo osteogenic differentiation via the p38 or the p44/42 MAPK pathways. The BMMSCs potentially undergo osteogenesis via p38 resulting in the up-regulation of OPN and the alizarin red content, thus demonstrating an efficient differentiation process. Comparatively, the AdMSCs could potentially differentiate via p44/42 which leads to an up-regulation of BMP-7 and thus the cells can differentiate into osteocytes, chondrocytes or adipocytes. As a result, the osteogenic differentiation is not as efficient and the numbers of osteocytes and mineralized nodules are much lower than that in BMMSCs. Our proposed model to describe these variations in BM and ADMSCs is illustrated in Fig. 8.

## Conclusion

In conclusion, the adult MSCs isolated from goat bone marrow and adipose tissue exhibit differences in their proliferation and osteogenic differentiation when they are cultured *in vitro*. These differences are in part, due to the source and passaging of MSCs, and the distinct signaling pathways that the MSCs go through during differentiation.

Serial passaging of MSCs from both sources results in reduced proliferation and osteogenic differentiation, the exact mechanism of which could not be determined from the reported data. The ADMSCs is an attractive source for MSCs but low passaged (<3) cells should be used for therapy. Comparatively, BMMSCs between passages 1–6 are acceptable. Osteogenesis in MSCs from both sources occurs using two distinct MAPK pathways. Understanding the putative signals during osteogenic differentiation may lead to the understanding of the osteogenic process that the MSCs undergo and also in the development of therapies, to achieve an optimal level of osteogenesis in the treatment of bone related defects.
